# Development and use of the Cytoscape app GFD-Net for measuring semantic dissimilarity of gene networks

**DOI:** 10.12688/f1000research.4573.1

**Published:** 2014-07-01

**Authors:** Juan J. Diaz-Montana, Norberto Diaz-Diaz

**Affiliations:** 1 School of Engineering, Pablo de Olavide University, Seville, 41013, Spain

## Abstract

Gene networks are one of the main computational models used to study the interaction between different elements during biological processes being widely used to represent gene–gene, or protein–protein interaction complexes. We present GFD-Net, a Cytoscape app for visualizing and analyzing the functional dissimilarity of gene networks.

## Introduction

The avalanche of information that scientists have faced during the last few years in the “-omics” fields, has made it essential to have an appropriate computational model to run automated analysis on huge datasets ^1^. Gene networks have arisen as a straightforward way of representing the interaction between different elements during biological processes. Gene-gene and protein-protein interaction networks have become a widely accepted way of studying how sets of proteins participate together in different biological processes ^2^, and multiple inference methods have been developed during the past years ^3– 6^. However, those inferred networks must be validated in order to verify their quality and reliability.

GFD-Net provides a novel approach to assessing the functional dissimilarity of a gene network, i.e. the degree of dissimilarity between its genes, taking into account the relationships between them defined by the network topology. As is well known, genes may have more than one function in the organism. GFD-Net is based on an adaptation of GFD ^7^. It uses Gene Ontology (GO) ^8^ in order to find the most cohesive (common and specific) function of each gene based on the overall performance of the entire network. Then, it weighs each edge according to the dissimilarity between the two nodes, i.e. how close their selected functions are, and calculates a numerical value of the dissimilarity of the whole network. This value reveals the "goodness" or "quality" of the network and shows in which way the genes are closer to each other according to the information contained in GO, helping researchers to identify the overall function of the network and how each gene participates in it.

Currently, there are two main approaches for gene network validation: a direct comparison between the inferred network with gene-gene interaction repositories ^9^ and gene annotations of biological entities ^10^. At present there are different techniques to analyze the semantic similarity of a set of genes or gene-products ^11^. However, to the authors’ knowledge, none of them take into account how such genes are related to each other. GFD-Net provides a new approach that also takes into account the network topology and has the advantage of constant improvement, as more specific terms are added to GO over time.

GFD-Net has been integrated in Cytoscape ^12^ as a plugin (versions 2) and as an app (versions 3). Cytoscape is a software platform for the visualization and analysis of networks, specializing in biological networks. It provides a user-friendly interface which allows users with limited software programming knowledge to use complex algorithms and computational techniques. It also has a wide range of apps ^13^ which provide the user with the opportunity to obtain or modify a gene network using any existing app and then analyze it using GFD-Net. The large user base of Cytoscape and its apps provides the latter with a much higher visibility within the research community than they would have if they were released as stand-alone programs.

In this paper, we present the implementation of GFD-Net app for Cytoscape 3 and two simple use cases.

## Implementation

GFD-Net is implemented in java and its only dependency is a JDBC driver which allows it to connect to the Gene Ontology database.

### Workflow

Firstly, GFD-Net provides different dialogs to configure the database connection details (url, user and password), the ontology to use during the analysis, and the organism to which the network being analyzed belongs to.

Next, the Cytoscape network is parsed and stored in memory using our own optimized structure for searching and quick access. The gene products associated to each gene are retrieved according to the Entrez database ^14^, the relevant GO-terms, and the relevant section of the GO-Tree ^15^ are loaded. Each of the proteins can be associated with, or located in one or more cellular components and be active in one or more biological processes where it can perform several molecular functions. Each annotation is represented in GO by a GO-term.

GFD-Net then computes all the possible combinations of GO-terms associated to each gene in the network and tries to find the most cohesive one. Next, each edge is weighted by the dissimilarity between the selected GO-terms for the nodes at its ends, and the whole network is weighted by the average of the edge weights. Both the weights and the network dissimilarity values range from 0 to 1, where 0 and 1 represent the best and the worst values respectively.

Finally, in order to facilitate the user’s interaction with the information retrieved, a result panel is displayed on the right side allowing the user to visualize all the obtained information by simply interacting with the network or the panel itself. The results are displayed in a way that allows the user to get general information about the network, or more specific information about each relationship or gene.

More details about how GFD-Net works can be found on the GFD-Net website: http://juanjoDiaz.github.com/gfdnet.

### Architecture

Originally, GFD-Net was a Cytoscape 2 plugin, but as soon as Cytoscape 3 was launched we ported it to an app following the Simple App approach which uses the app API to make the development similar to the old plugins. This approach requires no knowledge of the Cytoscape 3 architecture and allows a plugin to be ported with a minimal number of changes in the code but presents the same issues existing on Cytoscape 2 and its plugins. For this reason, we ported the code to a Bundle app better exploiting the benefits of the new architecture based on OSGi microservices ^16^ and relying on Maven ^17^ for dependency control and build instructions.

**Figure 1.**
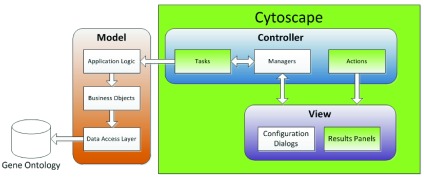
Diagram of GFD-Net architecture. The areas in green are directly extending or using the Cytoscape API.

GFD-Net is built following the mediating-controller MVC architecture which modularizes the code better, simplifying the maintainability of the project. By using this architecture, the app can be updated easily. For example, if the Gene Ontology database changes, or we decide to offer GFD-Net as a web service using Cytoscape.js only the data access layer or the view layer respectively will need to be modified. Figure 1 provides an overview of GFD-Net architecture.

The Model is completely independent of Cytoscape. It contains the application logic, the business objects and the data access layer. Since we need to traverse through a section of the GO-Tree that might be fairly large, the main challenge during the development of GFD-Net was the performance of the app. Thus, the data access layer is implemented so all the data extracted from the database is cached in memory to avoid redundant calls to the database. Furthermore, all the objects and structures used are optimized for minimal memory usage and quick searches. The retrieved data, such as genes, gene-products, GO-terms, etc., is cached in sorted sets so there are no duplicates and a specific element can be found quickly by using a binary search when needed.

The View is the layer that relies most heavily on Cytoscape’s swing application API. On the network views provided by Cytoscape the viewmodel API is used to hide or show nodes as necessary, and the model API events are used to capture the user interactions. The extensions that Cytoscape add are built using Swing and divided in two groups. The configuration dialogs are plain JDialog and provide a user-friendly interface to configure GFD-Net. The results panels are JPanels implementing the CytoPanelComponent interface in order to integrate the GFD-Net Panels in the Cytoscape UI.

The Controller gets notified of changes in the views, makes the necessary calls to the model and updates the views accordingly, completely decoupling the View from the Model. It contains actions, managers and tasks. The actions extend the AbstractCyAction class provided by the swing application API to display the menus and buttons. The managers control the different aspects of the application. There are managers to control the toolbar buttons (through the actions), the results panels, the network interactions and the core algorithm. They create the different views when necessary and are notified of user gestures on the View. Finally, the manager needs to communicate with the model to perform different operations or retrieve the content of the views. On Java Swing, everything that happens through an event (clicking a button, pressing a key, etc.) is processed by the event dispatcher thread. This means that any other event will be stuck until the current process ends and the whole UI will be blocked. Tasks extending the AbstractTask class provided by the work API of Cytoscape are run in secondary threads avoiding this issue when long running tasks are executed. Of course not all our tasks take long enough to make it necessary to use a task, so some of the calls to the model are done directly to the model. Tasks are especially important when preloading an organism (see GFD-Net website) or running the GFD-Net algorithm. Both processes can be slow (2–3 min.). GFD-Net disables all its buttons during task executions to avoid user modifications to the parameters while the program is working.

## Results

GFD-Net provides an intuitive way of running a functional dissimilarity analysis on a gene network. It can be found in the Apps menu, and in order to get started, a network should already be loaded; otherwise an error will be displayed. GFD-Net adds buttons to the Cytoscape toolbar to configure the database connection, set the ontology, set the organism (preloading it or not), run an analysis and refresh the app loading the current network as selected. These buttons open the different configuration dialogs which are very user-friendly and do not require any additional details. Once all the parameters have been set, clicking on the execute button starts the analysis. When the analysis is completed, a tabbed panel showing the results is displayed on the right.

In order to show the usefulness of GFD-Net, we have analyzed two networks extracted from human pathways from Kegg ^18^ using Graphite ^19^; a tool found in the Bioconductor R package. Both networks can be found in the Dataset as plain text files. In both cases we configured GFD-Net the same way: online GO database (release of May 2014), Biological Process ontology and Homo Sapiens organism (without preload).

First, we analyzed the “Cardiac muscle contraction” pathway and obtained a dissimilarity value of 0.06 (see Cardiac muscle contraction analysis results summary in the Dataset) confirming that the network has a very high functional similarity. Looking into the GO-terms associated with each gene (see Cardiac muscle contraction analysis results summary in the Dataset), we can find that the same annotation, *GO:0030049* (muscle filament sliding), has been selected for all the nodes, and that many of them have annotations related to cardiac processes. It is important to note that the selected function is directly related to the pathway being evaluated proving the benefits of selecting the most cohesive set of input annotations in order to find what a networks does in the organism.

**Figure 2.**
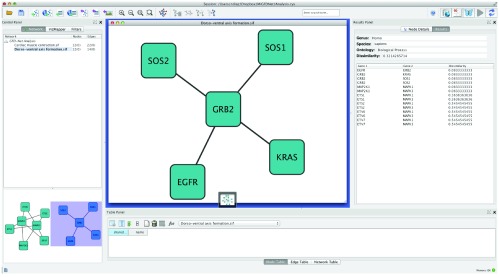
Screenshot showing what the default result panel looks like. It shows how the more specific genes are highly related while the more generic ones are not.

Then, we analyzed the “Dorso-ventral axis formation” and obtained a dissimilarity value of 0.32 (see Dorso-ventral axis formation analysis results summary in the Dataset). At first sight, this value might not be as low as expected but the results panel in Figure 2 or in the Dorso-ventral axis formation analysis results summary in the Dataset explains the reason. The network is divided in two sub-networks (see Figure 2). The one containing SOS1, SOS2, GRB2, EGFR and KRAS is highly cohesive and all its genes have the same annotation selected, *GO:0007411* (axon guidance), which is directly related with the pathway. The second one contains the nodes MAPK1, MAP2K1, MAPK3 which also have selected *GO:0007411*, but also ETS1 which has selected *GO:0048870* (cell motility) and ETS2, ETV6 and ETV7 which have selected *GO:0030154* (cell differentiation). The two later annotations show more generic functions and do not add much information about the network function, producing a higher dissimilarity.

Dataset 1. GFD-Net use cases Dataset http://dx.doi.org/10.5256/f1000research.4573.d30437
Cardiac muscle contraction gene network
Gene network extracted using Graphite from the pathway in Kegg.
Cardiac muscle contraction analysis results summary
It shows the dissimilarity of the whole network, the GO-Term selected for each gene and the dissimilarity of each edge as they are shown in the results panel.
Dorso-ventral axis formation gene network
Gene network extracted using Graphite from the pathway in Kegg.
Dorso-ventral axis formation analysis results summary
It shows the dissimilarity of the whole network, the GO-Term selected for each gene and the dissimilarity of each edge as they are shown in the results panel.

## Conclusions

We have developed GFD-Net, a Cytoscape app that allows evaluating gene networks by finding the most common function among its genes, weighting of its edges and obtaining a value of is functional dissimilarity, as well as providing an easy way to visualize the results. As a Cytoscape app, it has the advantageous ability to interact with the broad range of existing apps. In addition, it is worth noting that GFD-Net will improve over time as more specific terms are added to gene ontology.

We have shown here, how GFD-Net provides researchers with an easy way to validate their inferred networks and find out in which way the genes in a network are related to each other. This information helps finding high functionally related subsets as well as the function of a specific gene in a given network.

Looking forward, it is important to note that GFD-Net is not only restricted to being used for evaluating existing networks, but it can also be used in a gene network inference algorithm to extract more accurate models. In this line, we would expose some of the methods of GFD-Net as an API so we can have multiple apps, or multiple algorithms incorporating it. It is also in our plans to add methods to use GFD-Net directly from the Cytoscape command line. In this way we could run Cytoscape headlessly and use it as backend for a Cytoscape.js ^20^-based website offering GFD-Net as a service.

## Data and software availability

F1000Research: Dataset 1. GFD-Net use cases Dataset, 10.5256/f1000research.4573.d30437 ^23^

**Software available from:**

**App store** http://apps.cytoscape.org/apps/gfdnet

**App website** http://juanjoDiaz.github.com/gfdnet

**Latest source code** https://github.com/juanjoDiaz/gfdnet

**Source code as at the time of publication** https://github.com/F1000Research/gfdnet

**Archived source code as at the time of publication** http://dx.doi.org/10.5281/zenodo.10625 ^24^

**License** Apache License, Version 2.0
