# Supplementary material for: Development and use of the Cytoscape app GFD-Net for measuring semantic dissimilarity of gene networks
Source: F1000Res. 2014 Jul 1;3:142. [Version 1] doi: 10.12688/f1000research.4573.1 (PMC4224201; doi:10.12688/f1000research.4573.1)
Supplement: Peer review history

# Peer review: Referee response for version 1

**DOI:** 10.5256/f1000research.4892.r6388

**Published:** 2014-11-03

**Authors:** Alexander Pico

**Affiliations:** 1 Gladstone Institutes, San Francisco, USA

The authors describe the latest port and usage of GFD-Net as a Cytoscape 3 app. The calculation of GO-based functional dissimilarity (GFD) on networks provides a useful way to assess and annotate inferred networks. As part of the calculation, each pairwise interaction is weighted, providing a more granular assessment of a given network. The app takes care of mapping from gene identifiers to GO terms, the GFD calculation and the interactive display of results. The authors also share their future plans to expose an API so other apps can call on GFD-Net as a service. A welcome idea.

I particularly appreciated the thorough Architecture section. Together with the open source code availability, this description will be helpful to future Cytoscape app developers interested in network model query performance, accessing GO resources and overall app design.

A minor suggestion to include in your next revision of the paper: The programming language, Java, should be capitalized (first sentence in Implementation).

I have read this submission. I believe that I have an appropriate level of expertise to confirm that it is of an acceptable scientific standard.

# Peer review: Referee response for version 1

**DOI:** 10.5256/f1000research.4892.r5760

**Published:** 2014-10-10

**Authors:** Cristina Rubino Escudero

**Affiliations:** 1 School of Computer Engineering, University of Seville, Seville, Spain

This paper describes the design, implementation and use of GFD-Net, a tool to assess the functional dissimilarity of a gene network and visualize information about the function of each gene in the network.

Overall, the paper is well written and provides a sound improvement on quality scoring of inferred gene networks. The abstract and keywords are appropriate and the workflow is clear. The architecture section provides useful information about how the different APIs provided by Cytoscape are use to integrate the app in Cytoscape. Finally, the use cases are well presented, easily reproducible and are a good proof-of-concept for picking most cohesive functions, proving how useful the tool can be by hinting some potential usages of this app in real biological problems.

As it is mentioned in the conclusion, I think that GFD-Net full potential can be unveiled by exposing the core algorithm as an API so other apps can use it in order to extract information or as a fitness function.

I have read this submission. I believe that I have an appropriate level of expertise to confirm that it is of an acceptable scientific standard.
